# Preventive Effects of Omega-3 and Omega-6 Fatty Acids on Peroxide Mediated Oxidative Stress Responses in Primary Human Trabecular Meshwork Cells

**DOI:** 10.1371/journal.pone.0031340

**Published:** 2012-02-03

**Authors:** Theofilos Tourtas, Marco T. Birke, Friedrich E. Kruse, Ulrich-Christoph Welge-Lüssen, Kerstin Birke

**Affiliations:** Department of Ophthalmology, University Hospital Erlangen, Erlangen, Germany; University of Nebraska Medical Center, United States of America

## Abstract

Pathologic processes in glaucoma include increased apoptosis, accumulation of extracellular material in the trabecular meshwork and optic nerve, condensations of the cytoskeleton and precocious cellular senescence. Oxidative stress was shown to generate these alterations in primary ocular cells. Fatty acids omega-3 and -6 are alleged to constitute a prophylaxis against these deleterious effects. Here, we tested actual preventive effects omega-3 and -6 against peroxide induced stress responses in primary human trabecular meshwork cells. Changes of mitochondrial activity, proliferation, heat shock proteins, extracellular matrix components, and inflammatory markers were evaluated. Alterations of the cytoskeleton were evaluated by phalloidin labeling. Here we report a repressive effect of omega-6 on metabolic activity and proliferation, which was not detected for omega-3. Both agents were able to prevent the anti-proliferative effect of H_2_O_2_, but only omega-3 prevented metabolic repression. Expression of heat shock protein 27 was unaltered by both fatty acids, whereas heat shock protein 90 was significantly induced by both. Omega-6 increased fibronectin and connective tissue growth factor synthesis, as well as the amount of secreted fibronectin. Omega-3, instead, induced plasminogen activator inhibitor 1 synthesis. H_2_O_2_ further increased fibronectin production in omega-6 supplemented cells, which was not the case in omega-3 treated cells. H_2_O_2_ stimulation of plasminogen activator inhibitor 1 and connective tissue growth factor was repressed by both fatty acids. Both fatty acids appeared to abolish H_2_O_2_ mediated stimulation of nuclear factor κB and IL-6, but not IL-1α and IL-8. H_2_O_2_ induced formation of cross-linked actin networks and stress fibers, which was reduced by preemptive application of omega-3. Omega-6, in contrast, had no protective effect on that, and even seemed to promote condensation. Based on the observed side effects of omega-6, omega-3 appears to be the more beneficial fatty acid in respect of prophylactic intake for prevention of a glaucomatous disease.

## Introduction

The trabecular meshwork (TM) accounts for about 70–90% of total aqueous humor (AH) outflow from the anterior chamber in the adult human eye. Moreover, the TM constitutes an outflow resistance and thereby determines the intraocular pressure (IOP). Increase of outflow resistance and IOP, eventually, is a major risk factor in primary open angle glaucoma (POAG) [1]. POAG patients exhibit specific morphological peculiarities as accumulations of extracellular matrix material (ECM) and condensation or clustering of the cytoskeleton. Frequently, signs of subclinical inflammation are reported. Moreover, deregulations on the cellular level affecting signaling pathways regulating apoptosis, cellular senescence and cell cycle control were observed in ocular cells of POAG patients [1], [2], [3], [4].

In vitro, such glaucoma-characteristic alterations were detected due to artificially induced oxidative stress in cultured human TM cells (hTM) [5], [6], [7], [8]. This lead to the hypothesis that oxidative stress might either be responsible or at least involved in the onset and progression of the TM changes observed in POAG. Supporting that, evidence for a higher frequency of impaired mitochondrial function has been reported in POAG patients [8], [9], [10], [11]. Tanwar et al. (2010) identified conspicuous variations in the mitochondrial DNA of patients with primary congenital glaucoma affecting the respiratory chain and leading to increased production of reactive oxygen species (ROS) [12]. Izzotti et al. (2003) found a deletion mutation of the glutathione S-transferase Mu 1 (GSTM1) gene in POAG patients which correlated with an increase of 8-hydroxydeoxyguanosine (8-OH-dG), a marker of DNA damage and precocious senescence, in TM cells [13]. Sacca et al. (2005) described a distinct correlation between oxidative DNA damage in the TM, visual field reduction and IOP increase [14]. Previously, Abu-Amero et al. (2006) had already observed a significant reduction of the mitochondrial respiratory activity in patients with POAG [9]. Mitochondrial dysfunctions and a reduced mitochondrial respiratory activity favor accumulation of ROS. Studies to evaluate the total reactive anti-oxidative potential of the aqueous humor state a significantly decreased anti-oxidative capacity in patients with POAG [15], [16], [17], [18]. All these findings suggest a constantly heightened oxidative stress level in patients with POAG [1]. Consequently, the role of oxidative stress in the pathogenesis of POAG has become focus of experimental studies [14], [18], [19], [20] and became a potential new target for therapeutic approaches.

In this context, preemptive application of dietary supplements with alleged preventive capacities from oxidative stress was proposed in terms of a prophylaxis or even therapy of ocular degenerative diseases [21]. Amongst these supplements, essential fatty acids omega (ω)-6 and ω-3 are of special interest due to their reported anti-inflammatory, antithrombotic, hypolipidemic, and vasodilatory capacities [22], [23]. There is already consistent evidence that ω-3 fatty acids are protective agents against ischemia-, light-, oxygen-, inflammation-, and age-associated pathologies of the vascular and neural retina [24], [25]. But there are also reports that indicate the requirement of a fine-tuned balance of fatty acid intake, as a misbalanced ω-3/ ω-6 ratio or excessive amounts of ω-6 fatty acids are suspected to promote cardiovascular, inflammatory and autoimmune diseases or cancer [26], [27], [28].

In the presented study, the effects of ω-6 and ω-3 fatty acids and their preventing capacities against oxidative stress induced glaucoma-associated expression changes were analyzed in cultivated hTM, the most vulnerable ocular tissue to oxidative stress [7], [11], [29].

## Materials and Methods

### Primary hTM cell culture and treatment

Explant cultures of hTM were obtained from the eye bank of the Ludwig-Maximilians-University, Munich, Germany. Methods of securing human tissue were humane, included proper consent and approval, complied with the Declaration of Helsinki and were approved by the ethic committee of the Department of Medicine of the Friedrich-Alexander-University Erlangen-Nuremberg. The consent statement was written (EK_No. 4346-CH). Monolayer cultures were established from eyes obtained 4 to 8 hours post mortem of five human donors (40–50 years) without any history of eye diseases. Cells were propagated in complete F10 (Ham's F10 medium supplemented with 10% fetal bovine serum [FBS], 10 U/ml penicillin, 10 µg/ml streptomycin, and 0.25 µg/ml Fungizone Mix; all from PAN™ Biotech GmbH, Aidenbach, Germany) under standard cell culture conditions in 6-well (RNA/ protein extraction) or 24 well (CCK-8/BrdU/IF-labeling) cell culture plates (Techno Plastic Products AG, Trasadingen, Switzerland).

To test the effects of ω-3 or ω-6 fatty acids, confluent hTM of passages 3 to 5 were pre-incubated in low F10 (Ham's F10/1% FBS) for 24 hours. Then the medium was substituted by low F-10 supplemented to nontoxic 50 µM ω-3 [30] or 16 µM ω-6 [31] fatty acids (both from Sigma-Aldrich, Taufkirchen, Germany). After 24 hours, medium was replaced by fresh ω-3 or ω-6 containing medium for an additional 24 hours incubation. After 48 hours in total, oxidative stress was induced by exposure to nontoxic 300 µM hydrogen peroxide [6] (H_2_O_2_, Sigma-Aldrich) for 1 hour. Afterwards the cells were washed with PBS and further cultured with the distinct media for 1 hour. In control cultures, the medium was changed at the same time points, but no H_2_O_2_ was added.

### Cell counting kit-8

Mitochondrial metabolism was quantified at 0, 24 and 48 hours after indicated treatments with a Cell Counting Kit-8 (CCK-8, Dojindo Molecular Technologies, Rockville, MD) according to the manufactures' instructions. 100 µl aliquots of the medium were transferred to 96 well plates and absorbance at 450 nm was measured with a spectrophotometer (Multiscan® Spectrum; Thermo Electron Corporation, Karlsruhe, Germany). Measurements were done as triplicates of hTM from 5 different donors in 3 independent experiments. Values represent mean averages ± SD (n = 45).

### BrdU incorporation

hTM proliferation was quantified with a Bromodeoxyuridine (BrdU) detection kit (Cell Proliferation ELISA, BrdU [colorimetric]; Roche Diagnostics, Mannheim, Germany). Therefore, 1 µM BrdU labeling solution was added to the cells during treatment for 48 hours. Detection was done according to the manufactures' instructions. Absorbance was measured at 370 nm wavelength with a spectrophotometer (Multiscan® Spectrum; Thermo). The tests were done as triplicates of hTM from 5 different donors in 3 independent experiments. Values represent mean averages ± SD (n = 45).

### RNA isolation and real-time PCR

Total RNA was isolated with a RNA isolation kit (RNeasy Fibrous Tissue Mini Kit; Qiagen N.V., Hilden, Germany) according to the manufactures' instructions. Structural integrity of the RNA samples was confirmed by electrophoresis in 1% Tris-acetate-EDTA (TAE)-agarose gels [32]. Yield and purity were determined photometrically. 200 ng of mRNA were transcribed to cDNA by reverse transcription using a reverse transcription-PCR kit (Access RT-PCR Introductory System; Promega Corporation, Madison, USA). Real-time PCR quantification was performed in 40 cycles in a thermocycler (LightCycler System; Roche Diagnostics, Penzberg, Germany). The selected primers for FN, PAI-1, CTGF, Hsp27, Hsp90, IL-1α, IL-6, IL-8, NFκB and glycerinaldehyd-3-phosphat-dehydrogenase (GAPDH) were purchased from Metabion (Metabion International AG, Martinsried, Germany); primer sequences are summarized in Table 1. Corresponding probes were selected with the ProbeFinder v2.04 software (Roche). The standard curve was obtained from probes of three different untreated hTM cultures. As internal control GAPDH was processed simultaneously in each assay and levels of FN-1, PAI-1, CTGF, Hsp27, Hsp90, IL-1α, IL-6, IL-8 and NFκB mRNAs were determined as relative ratios (RR) by division by GAPDH. Ratios in non-supplemented cells without H_2_O_2_ treatment were set to one and expression levels of treated cells are given as fold of that. All experiments were performed in triplicates with TM cultures from three different donors. Values represent mean averages ± SD (n = 9).

**Table 1:** Primers used for realtime PCR.

Gene	Sequence	Probe
Hsp27	fwd.: 5′-tgacggtcaagaccaagga-3′	22
	rev.: 5′-tgtagccatgctcgtcctg-3′	
Hsp90	fwd.: 5′-ggagaattctacaagagcctcact-3′	48
	rev.: 5′-tgaccttctacagaaaagtgcttg-3′	
FN	fwd.: 5′-ctggccgaaaatacattgtaaa-3′	32
	rev.: 5′-ccacagtcgggtcaggag-3′	
PAI-1	fwd.: 5′-aaggcacctctgagaacttca-3′	19
	rev.: 5′-cccaggactaggcaggtg-3′	
CTGF	fwd.: 5′-ctgcaggctagagaagcagag -3′	85
	rev.: 5′-gatgcactttttgcccttct-3′	
IL-1α	fwd.: 5′-acaaaaggcgaagaagactga-3′	20
	rev.: 5′-ggaactttggccatcttgac-3′	
IL-6	fwd.: 5′-caggagcccagctatgaact-3′	45
	rev.: 5′-gaaggcagcaggcaacac-3′	
IL-8	fwd.: 5′-agacagcagagcacacaagc-3′	72
	rev.: 5′-atggttccttccggtggt-3′	
NFκB	fwd.: 5′-cgggatggcttctatgagg-3′	47
	rev.: 5′-ctccaggtcccgcttctt-3′	
GAPDH	fwd.: 5′-agccacatcgctcagacac-3′	60
	rev.: 5′-gcccaatacgaccaaatcc-3′	

### Protein isolation and western blot

Media of hTM were collected separately and concentrated six fold by centrifugation (Vivaspin20; Sartorius Stedim GmbH, Goettingen, Germany). Cells were directly lysed in RIPA buffer (150 mM NaCl, 1% NP-40, 0.5% DOC, 0.1% SDS, 50 mM Tris [pH 8.0], 4 mM DTT, 0.5 mM NaVanadate, 2 mM NaF, 2 mM phenylmethylsulfonyl fluoride) containing protease inhibitors (complete protease inhibitor cocktail; Roche). Protein contents of concentrated media and cell lysates were determined by the bicinchoninic acid (BCA) protein assay (Pierce, Rockford, USA). Samples were supplemented with one fourth SDS-loading buffer (Roti-load-1; Roth, Karlsruhe, Germany) and aliquots containing equal proteins were separated by SDS-polyacrylamide gel electrophoresis (PAGE). Proteins were transferred onto a nitrocellulose membrane (Protran Ba-183; Whatman, Dassel, Germany) by semi-dry or tank blotting. Further procedure was done as previously described [33] and primary antibodies (Table 2) were added over night at 4°C. Corresponding secondary alkaline phosphatase (AP) -conjugated antibodies (Table 2) were incubated for 30 minutes at room temperature. After substrate incubation (CDP-star; Roche) the signals were visualized by exposure to light sensitive films (Hyperfilm ECL; GE Healthcare, Munich, Germany), which were digitized and densitometrically quantified with the Multi Gauge V3.1 software (Fujifilm, Duesseldorf, Germany). All experiments were performed in triplicates with hTM cultures from three different donors. Values represent mean averages ± SD (n = 9).

**Table 2:** Antibodies used for Western blots (WB) and Immunofluorescence (IF) labeling.

Antibody	Dilution/Application	Supplier
Rabbit monoclonal anti-human Hsp27	1∶1000 (WB)	Sigma-Aldrich
Mouse monoclonal anti-human Hsp90	1∶1000 (WB)	Sigma-Aldrich
Rabbit polyclonal anti-human FN	1∶1000 (WB)	St.Cruz
Rabbit polyclonal anti-human PAI-1	1∶500 (WB)	Abcam
Rabbit polyclonal anti-human CTGF	1∶1000 (WB)	Abcam
AP conjugated goat anti-mouse IgG	1∶10000 (WB)	Sigma-Aldrich
AP conjugated goat anti-rabbit IgG	1∶10000 (WB)	Sigma-Aldrich

### Fibronectin ELISA

Medium contents for FN were analyzed by ELISA according to the manufacturer's instructions (QuantiMatrix Human Fibronectin ELISA KIT; Millipore, Schwalbach, Germany). Aliquots of 50 µl of six-fold concentrated cell media were set in. Absorbance at 450 nm was measured with a spectrophotometer (Multiscan® Spectrum; Thermo). Measurements were done as triplicates of media from hTM cultures of 3 different donors in 3 independent experiments. Values represent mean averages ± SD (n = 9).

### Interleukin ELISAs

Amounts of secreted Interleukins (IL) -6 and -8 were analyzed by ELISA according to the manufacturer's instructions (QuantiGlo Chemiluminescent ELISA; R&D Systems, Minneapolis, USA). Aliquots of 100 µl and 50 µl of six-fold concentrated cell media were used for IL-6 and IL-8 determination, respectively. Light emission was measured with a luminometer (Fluoroskan Ascent FL; Thermo). Measurements were done as triplicates of hTM cell media from 3 different donors in 3 independent experiments. Values represent mean averages ± SD (n = 9).

### Nuclear Factor (NF) κB Assay

Nuclear content of NFκB was tested in nuclear extracts with a NoShift™ NFκB Transcription Factor Assay (Novagen/Merck, Darmstadt, Germany) according to the manufacturer's instructions. For nuclear extracts, cells were collected from plates with Trypsine/EDTA (PAN™), washed three times in Hank's buffered salt solution (HBBS; PAN™) and lysed in three times packed cell volumes of low-salt hypotonic cell lysis buffer (20 mM HEPES pH 7.5, 10 mM KCl, 5 mM MgCl2, 0.5 mM EDTA, 0.1% TritonX-100, 10% glycerol, protease inhibitor cocktail [Roche]) for 10 min on ice. Nuclei were pelleted by centrifugation for 10 sec at 4°C and cytosolic fractions (supernatants) were discarded. Nuclei were washed once in low-salt hypotonic cell lysis buffer, and extracted using high-salt hypotonic cell lysis buffer (low-salt supplemented to 500 mM NaCl) for 10 min on ice. Debris was sedimented by centrifugation for 30 min at 4°C and nuclear extracts were transferred to fresh vials. After BCA protein determination extracts were stored at −80°C until use. For assays, equal masses of proteins were set in. Quantification was done by measurement of the absorbance at 450 nm with a spectrophotometer (Multiscan® Spectrum; Thermo). Measurements were done as triplicates of nuclear extracts of hTM cell cultures from 3 different donors in 3 independent experiments. Values represent mean averages ± SD (n = 9).

### Immunofluorescence (IF) labeling

hTM were grown for 48 hours on microscope chamber slides (Lab-Tek®II; Nunc, Rochester, USA). After depicted treatments, slides were fixed in 4% paraformaldehyde (PFA), blocked in PBS (1% BSA, 0.1% Triton X-100) and primary antibodies (Table 2) were added in PBS (2% BSA, 0.2% Triton X-100) overnight at 4°C. Fluorophor conjugated secondary antibodies (Table 2) in PBS were added for 30 minutes at room temperature. The F-actin cytoskeleton was labeled by fluorescein conjugated phalloidin (Molecular Probes™, Eugene, USA) for 15 minutes at room temperature. Nuclei were counterstained by 4′,6′-diamidino-2-phenylindole (DAPI; Molecular Probes™). Cells were mounted in fluorescent mounting medium (Dako, Glostrup, Denmark) and analyzed by Laser confocal microscopy (Carl Zeiss, Goettingen, Germany). All experiments were performed in triplicates with hTM cultures from three different donors (n = 9).

### Statistical analysis

Statistical analysis was done by a Student's t-test with the GraphPad Prism5 software (*p≤0.05; **p≤0.01; ***p≤0.001).

## Results

### Mitochondrial activity analysis

**Figure 1:**
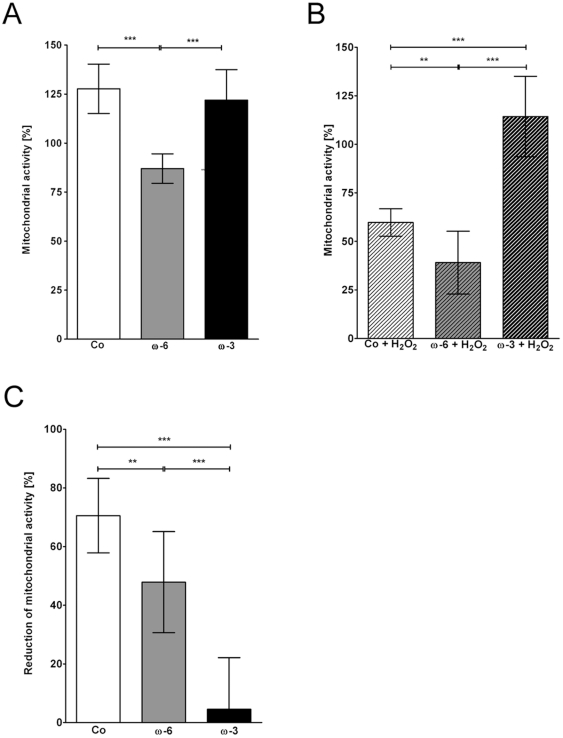
CCK-8 assay. Quantification of mitochondrial activity in hTM after 48 hours normalized to starting activity in controls. (**A**) Effects of ω-6 (16 µM) and ω-3 (50 µM) fatty acids compared to controls (Co). (**B**) Effects of H_2_O_2_ in controls, ω-6 and ω-3 fatty acids pre-treated hTM. (**C**) %-reduction of mitochondrial activity after H_2_O_2_ exposition. Values represent mean averages (m.a.) ± standard deviations (sd) of three independent experiments performed in triplicates of 5 different donors (n = 45); asterisks: p-values of statistical significances (**p≤0.01; ***p≤0.001).

Mitochondrial activity of primary hTM cultured under standard conditions (controls) increased to 130±11% over 48 hours (Fig. 1A). Cells supplemented with ω-3 showed a similar activity at the same time point (122±16%; *p* = 0.2211), whereas the mitochondrial activity of cells preincubated with ω-6 was significantly lower at 87±8% (***p≤0.001; Fig. 1A) compared to the controls.

Exposure to H_2_O_2_ significantly reduced mitochondrial activity of controls to 60±7% (***p≤0.001; Fig. 1B), corresponding to a 71±13% reduction (Fig. 1C). The mitochondrial activity of ω-6 preincubated, H_2_O_2_ stimulated hTM was 39±16% (***p≤0.001; Fig. 1B), corresponding to a 48±17% (Fig. 1C) reduction compared to the ω-6-only incubated hTM. hTM preincubated with ω-3 in contrast, showed no significant reduction due to H_2_O_2_ (114±21%; Fig. 1B; reduction 5±18% Fig. 1C). Compared to the controls H_2_O_2_ mediated reductions were significantly reduced in both, ω-6 (**p≤0.01) and ω-3 (***p≤0.001) preincubated hTM (Fig. 1C).

### Proliferation analysis

**Figure 2:**
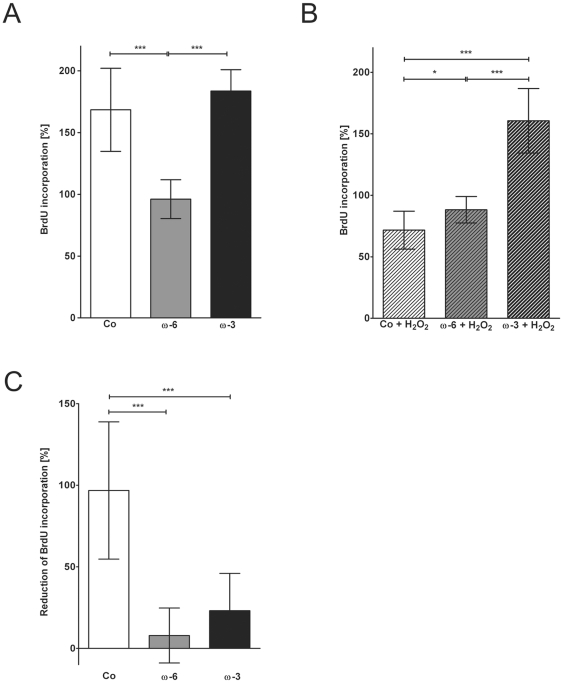
BrdU incorporation analysis. Quantification of proliferation rate in hTM after 48 hours normalized to starting activity in controls. (**A**) Effects of ω-6 (16 µM) and ω-3 (50 µM) fatty acids compared to controls (Co). (**B**) Effects of H_2_O_2_ in controls, ω-6 and ω-3 fatty acids pre-treated hTM. (**C**) %-reduction of BrdU-incorporation after H_2_O_2_ exposition. Values represent m.a. ± sd of three independent experiments performed in triplicates of 5 different donors (n = 45); asterisks: p-values of statistical significances (*p≤0.05; **p≤0.01; ***p≤0.001).

Controls had a mean BrdU incorporation rate of 168±34% after 48 hours normalized to t = 0 hours. Cells preincubated with ω-3 showed a similar increase of incorporation rate as the controls up to 184±17% at 48 hours (*p* = 0.2447). In contrast, ω-6 preincubated cells' incorporation rate remained rather constant over 48 hours reaching a final value of 96±16%, which was significantly lower than in the controls and the ω-3 supplemented cells (***p≤0.001; Fig. 2A).

H_2_O_2_ exposure reduced BrdU incorporation in controls by 97±42% to 72±15% (***p≤0.001; Fig. 2B). In cells preincubated with fatty acids the incorporation rates were not reduced by H_2_O_2_ and were in the same range as in the corresponding controls at 88±11% (ω-6) and 161±26% (ω-3; Fig. 2B). The corresponding reductions were 8±17% (ω-6) and 23±23% (ω-3) respectively, both being significantly lower than in the controls (***p≤0.001; Fig. 2C).

### Expression of heat shock proteins (Hsp)

**Figure 3:**
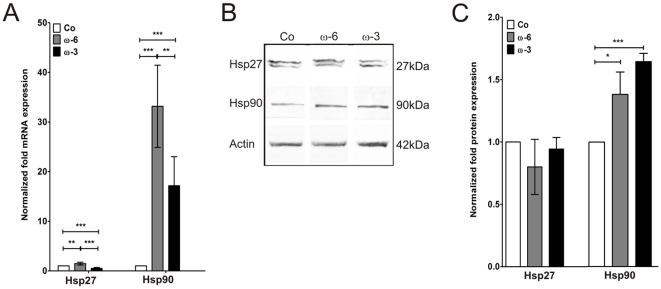
Hsp27 and Hsp90 expression analysis. (**A**) Quantification of realtime PCR expression analysis of Hsp27 and Hsp90 mRNAs in controls, ω-6 and ω-3 fatty acids pre-treated hTM normalized to controls. (**B**) Western blot detection of cellular Hsp27, Hsp90 and actin protein in controls, ω-6 and ω-3 fatty acids pre-treated hTM. (**C**) Plot of densitometric quantifications of Hsp27 and Hsp90 protein expression in controls, ω-6 and ω-3 fatty acids pre-treated hTM adjusted to actin expression and normalized to controls. Values represent m.a. ± sd of three independent experiments performed on cells of three different donors (n = 9); asterisks: p-values of statistical significances (*p≤0.05; **p≤0.01; ***p≤0.001).

Hsp27 mRNA levels were significantly increased after preincubation with ω-6 by 1.5±0.3 fold (**p≤0.01) normalized to the controls (Fig. 3A). Preincubation with ω-3, in contrast, significantly decreased the mRNA level by 0.5±0.2 fold (***p≤0.001; Fig. 3A). On protein level, however, amounts of Hsp27 were equal in controls and fatty acid preincubated cells (ω-6: 0.8±2.2 fold; *p* = 0.1923; ω-3: 0.9±0.1 fold; *p* = 0.3503; Fig. 3B, C).

Hsp90 mRNA was increased by both fatty acids by 33±8 fold (ω-6; ***p≤0.001) and 17±6 fold (ω-3; ***p≤0.001; Fig. 3A), respectively. Hsp90 protein levels were also increased by both fatty acids by 1.4±0.2 fold (ω-6; *p≤0.05) and 1.6±0.1 fold (ω-3; ***p≤0.001; Fig. 3B, C).

### Expression of ECM components

**Figure 4:**
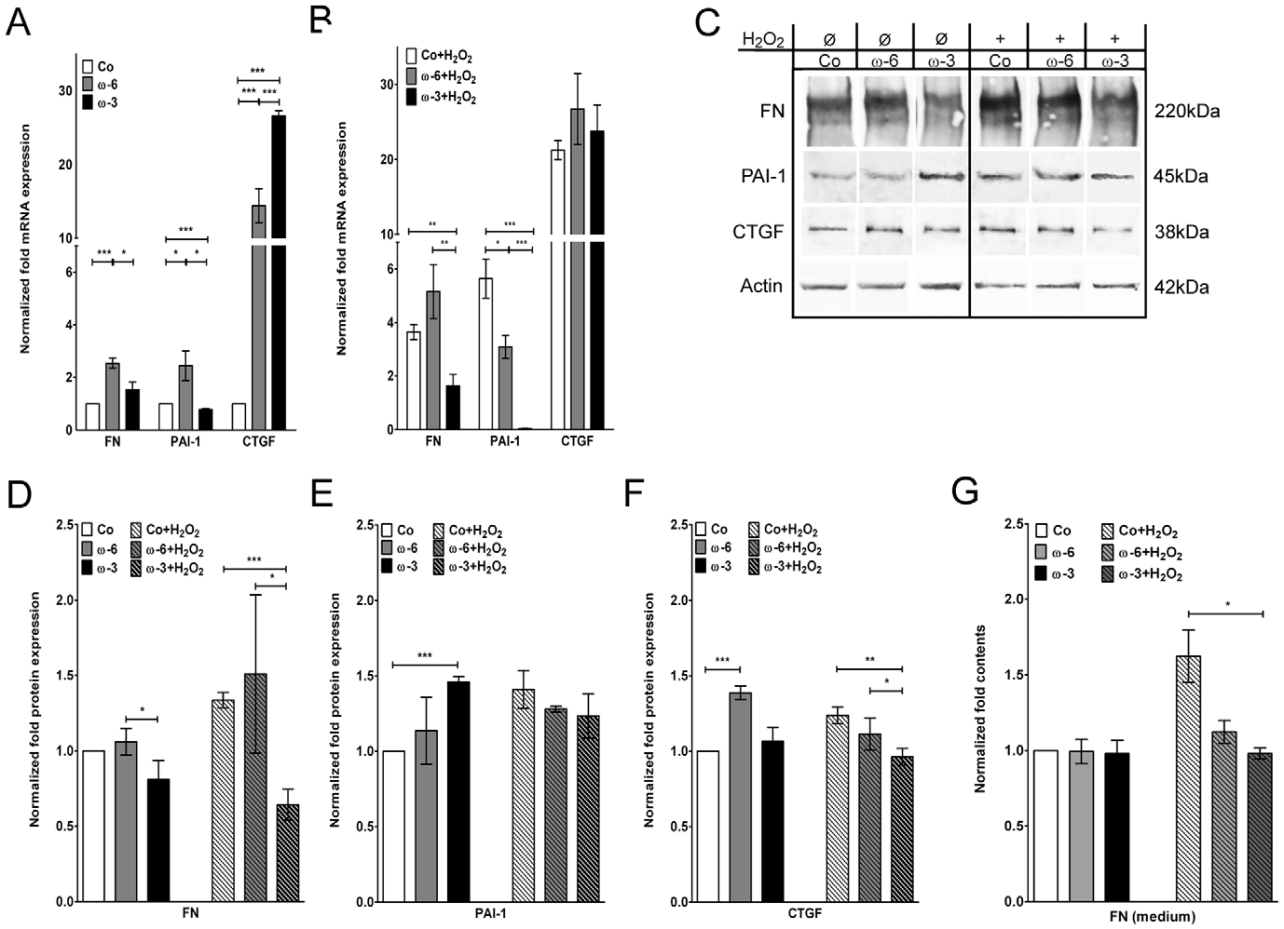
FN, PAI-1 and CTGF expression analysis. Quantitative realtime PCR expression analysis of FN, PAI-1 and CTGF mRNAs in controls, ω-6 and ω-3 fatty acids pre-treated hTM normalized to controls (**A**) before and (**B**) after H_2_O_2_ exposition. (**C**) Western blot detection of cellular FN, PAI-1, CTGF, and actin in controls, ω-6 and ω-3 fatty acids pre-treated hTM. Plots of densitometric quantifications to deduce fold expressions of intracellular (**D**) FN, (**E**) PAI-1 and (**F**) CTGF, before and after H_2_O_2_ exposition. (**G**) ELISA quantification of FN medium contents normalized to controls. Values represent m.a. folds ± sd of three independent experiments performed on cells of three different donors (n = 9); asterisks: *p*-values of statistical significances (*p≤0.05; **p≤0.01; ***p≤0.001).

ω-6 fatty acid significantly increased the fibronectin (FN) mRNA by 2.5±0.5 fold (***p≤0.001) compared to controls, while ω-3 supplementation did not have a significant effect (1.5±0.8 fold; *p* = 0.1158; Fig. 4A). Intracellular FN protein levels were not changed upon fatty acid supplementation (ω-6: 1.1±0.1 fold; *p* = 0.3073; ω-3: 0.8±0.1 fold; *p* = 0.0584; Fig. 4C, D), amounts of secreted FN in the medium were also not affected (ω-3: *p* = 0.8279; ω-6: *p* = 0.9382; Fig. 4G).

In the controls, H_2_O_2_ addition increased the FN mRNA by 3.7±0.7 fold (***p≤0.001; Fig. 4B), and the intracellular FN protein by 1.3±0.1 fold (***p≤0.001; Fig. 4C, D). In ω-6 pre-treated cells, H_2_O_2_ led to a further increase of FN mRNA to 5.2±2.5 fold (*p≤0.05), whereas the amount of intracellular FN protein did not increase (1.5±0.1 fold) compared to the corresponding controls (*p* = 0.2168; Fig. 4C, D). Pre-treatment with ω-3 prevented the H_2_O_2_ stimulation of FN mRNA (1.6±1.1 fold; *p* = 0.8420; Fig. 4B) and intracellular FN (0.6±0.1 fold; ***p≤0.001; Fig. 4C, D). In ELISA analysis H_2_O_2_ increased FN in the medium to 1.6±0.3 fold (*p≤0.05). Both fatty acids appeared to impede this stimulation, in case of ω-3 even to a statistically significant extent (*p≤0.05; ω-6: *p* = 0.0573; Fig. 4G).

Plasminogen activator inhibitor (PAI-)1 mRNA expression was significantly increased by 2.5±0.5 fold in ω-6 supplemented cells (*p≤0.05), whereas ω-3 reduced the expression level compared to the controls (0.8±0.1 fold; ***p≤0.001; Fig. 4A). On the protein level, in contrast, PAI-1 was significantly increased to 1.5±0.1 fold in ω-3 supplemented cells (***p≤0.001), while ω-6 did not affect PAI-1 protein levels (1.1±0.2 fold; *p* = 0.3462; Fig. 4C, E).

Exposure to H_2_O_2_ stimulated PAI-1 mRNA expression in the controls by 5.6±0.2 fold (***p≤0.001) and cellular PAI-1 protein levels were increased to 1.4±0.2 fold (**p≤0.01; Fig. 4C, E). In cells pre-treated with ω-6 fatty acid the PAI-1 mRNA was not further increased by H_2_O_2_ (3.1±1.1; *p* = 0.3824). The same applied for the PAI-1 protein levels, which remained constant after H_2_O_2_ stimulation (1.3±0.1 fold; *p* = 0.3276; Fig. 4C, E). In cells preincubated with ω-3 the PAI-1 mRNA expression even dropped to 0.05±0.02 fold after H_2_O_2_ addition (***p≤0.001; Fig. 4B), protein levels, however, were in the same range than in their corresponding controls (1.2±0.1 fold; *p* = 0.0598; Fig. 4C, E).

Connective tissue growth factor (CTGF) mRNA expression was significantly upregulated in cells supplemented with fatty acids compared to the controls (ω-6:14.4±5.7 fold; ***p≤0.001; ω-3: 26.6±1.7 fold; ***p≤0.001; Fig. 4A). The ω-6 mediated activation of CTGF was also observed on the protein level by a 1.4±0.05 fold increase (***p≤0.001). Protein levels in ω-3 treated cells were not increased (1.1±0.1 fold; *p* = 0.2721; Fig. 4C, F).

H_2_O_2_ addition induced expression of CTGF mRNA by 21.2±3.1 fold (***p≤0.001) in the controls. In ω-6 pre-treated cells, H_2_O_2_ further increased mRNA expression to 23.7±8.6 fold (*p≤0.05), which was not observed in ω-3 pre-treated cells (26.7±11.6; *p* = 0.4423; Fig. 4B). On the protein level, H_2_O_2_ resulted in a weak, but significant upregulation of CTGF in the controls (1.2±0.05 fold; **p≤0.01). In ω-6 pre-treated cells, H_2_O_2_ exposure led to a reduced protein level than in the corresponding controls (1.1±0.05 fold; **p≤0.01). In ω-3 pre-treated cells, H_2_O_2_ exposure had no effect on CTGF protein level (1.0±0.05 fold; *p* = 0.1670 Fig. 4C, F).

### Expression of Interleukins and NFκB

**Figure 5:**
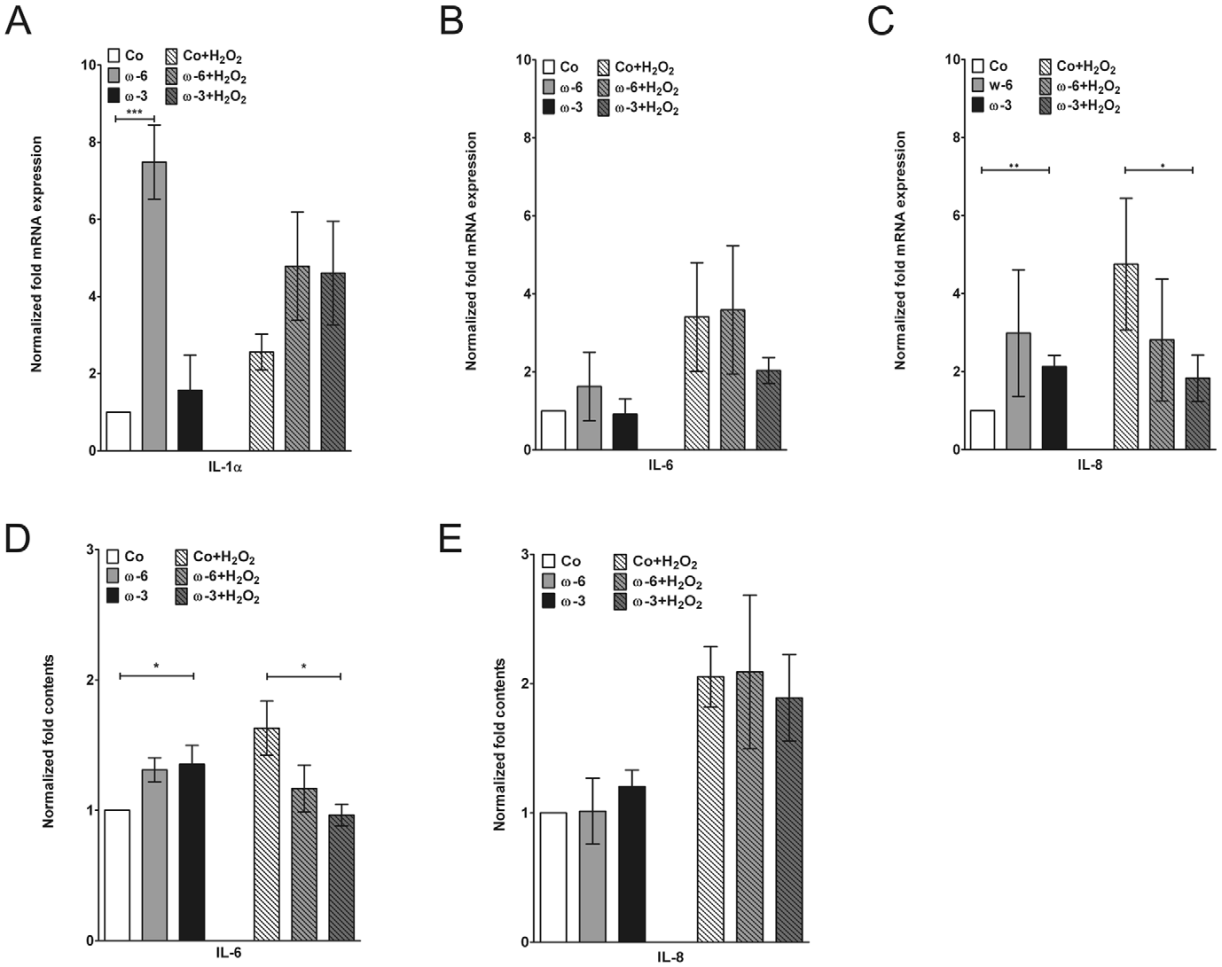
Interleukin 1α, -6 and -8 expression analysis. Quantitative realtime PCR expression analysis of interleukins (**A**) -1α, (**B**) -6 and (**C**) -8 mRNAs in controls, hTM pre-treated with ω-6 and ω-3 before and after H_2_O_2_ exposition. ELISA quantification of (**D**) IL-6 and (**E**) IL-8 medium contents. Values are normalized to untreated controls and represent m.a. folds ± sd of three independent experiments performed on cells of three different donors (n = 9); asterisks: *p*-values of statistical significances (*p≤0.05; **p≤0.01; ***p≤0.001).

Expression of IL-1α mRNA was significantly stimulated by ω-6 (7.5±1.0 fold; ***p≤0.001), whereas ω-3 had no effect (1.6±0.9 fold; *p* = 0.3408; Fig. 5A). Exposition to H_2_O_2_ doubled IL-1α mRNA expression (2.6±0.5 fold; **p≤0.01; Fig. 5A). Neither ω-3, nor ω-6 could significantly inhibited this stimulation (ω-3: *p* = 0.0669; ω-6: *p* = 0.0596; Fig. 5A). Expression in H_2_O_2_/ω-6 double treated cells appeared lower than in ω-6 supplemented hTM, differences, however, did not reach the level of statistical significance (*p* = 0.0513; Fig. 5A).

IL-6 mRNA expression was not altered by both fatty acids (ω-3: *p* = 0.7306; ω-6: *p* = 0.2828; Fig. 5B). H_2_O_2_ stimulation increased IL-6 mRNA to 3.4±1.4 fold (*p≤0.05), which could not be impeded by both fatty acids (ω-3: *p* = 0.1720; ω-6: *p* = 0.8920; Fig. 5B). ELISA analysis of the medium revealed that ω-6 stimulated IL-6 secretion (*p≤0.05) which was not observed upon ω-3 supplementation (*p* = 0.0791; Fig. 5D). H_2_O_2_ lead to a 1.6±0.3 fold induction of IL-6 protein (*p≤0.05; Fig. 5D). Both fatty acids appeared to reduce this stimulation, however, only the effect of ω-3 was significant (*p≤0.05; ω-6: *p* = 0.1675; Fig. 5D).

IL-8 mRNA expression appeared to be slightly activated by both fatty acids. However, this was statistically significant for ω-3 only (**p≤0.01; ω-6: *p* = 0.1017; Fig. 5C). Incubation with H_2_O_2_ increased IL-8 synthesis to 4.8±1.7 fold (*p≤0.05), which could be counteracted by ω-3 supplementation (*p≤0.05; Fig. 5C). Addition of ω-6 was inefficient here (*p* = 0.2174). Analysis of IL-8 in the cells medium indicated that fatty acids alone had no effect on IL-8 contents (ω-3: *p* = 0.0506; ω-6: *p* = 0.9324; Fig. 5E). Exposition to H_2_O_2_ led to a 2.0±0.2 fold increase of IL-8, which was statistically significant (**p≤0.01; Fig. 5E). With respect to H_2_O_2_ counteraction, both fatty acids were ineffective (ω-3: *p* = 0.5258; ω-6: *p* = 0.9256; Fig. 5E).

**Figure 6:**
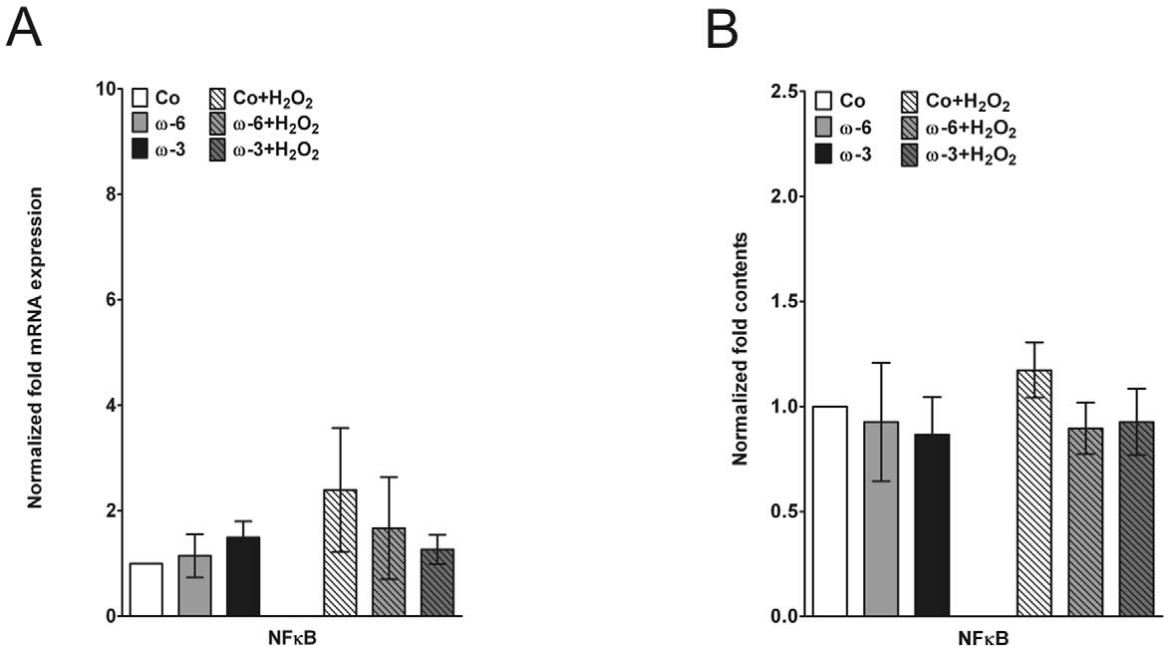
Analysis of nuclear NFκB. (**A**) Quantitative realtime PCR expression analysis of NFκB mRNA in controls, hTM pre-treated with ω-6 and ω-3 before and after H_2_O_2_ exposition. (**B**) Quantification of nuclear NFκB protein contents. Values are normalized to untreated controls and represent m.a. folds ± sd of three independent experiments performed on cells of three different donors (n = 9).

Realtime PCR analysis of NFκB expression did not reveal any significant influences of both fatty acids on this transcription factor (Fig. 6A). Data suggested that H_2_O_2_ slightly stimulated NFκB, but statistical significance was not reached (*p* = 0.1087; Fig. 6A). Notably, such an indication of increase was not detected in fatty acid supplemented hTM (Fig. 6A). On the protein level, a similar regulation was observed. Fatty acids alone did not affect the nuclear NFκB level, whereas H_2_O_2_, though insignificantly, stimulated nuclear contents (Fig. 6B). Again, such a slight increase was not observed in ω-3 or ω-6 supplemented hTM (Fig. 6B).

### Changes of the F-actin cytoskeleton

**Figure 7:**
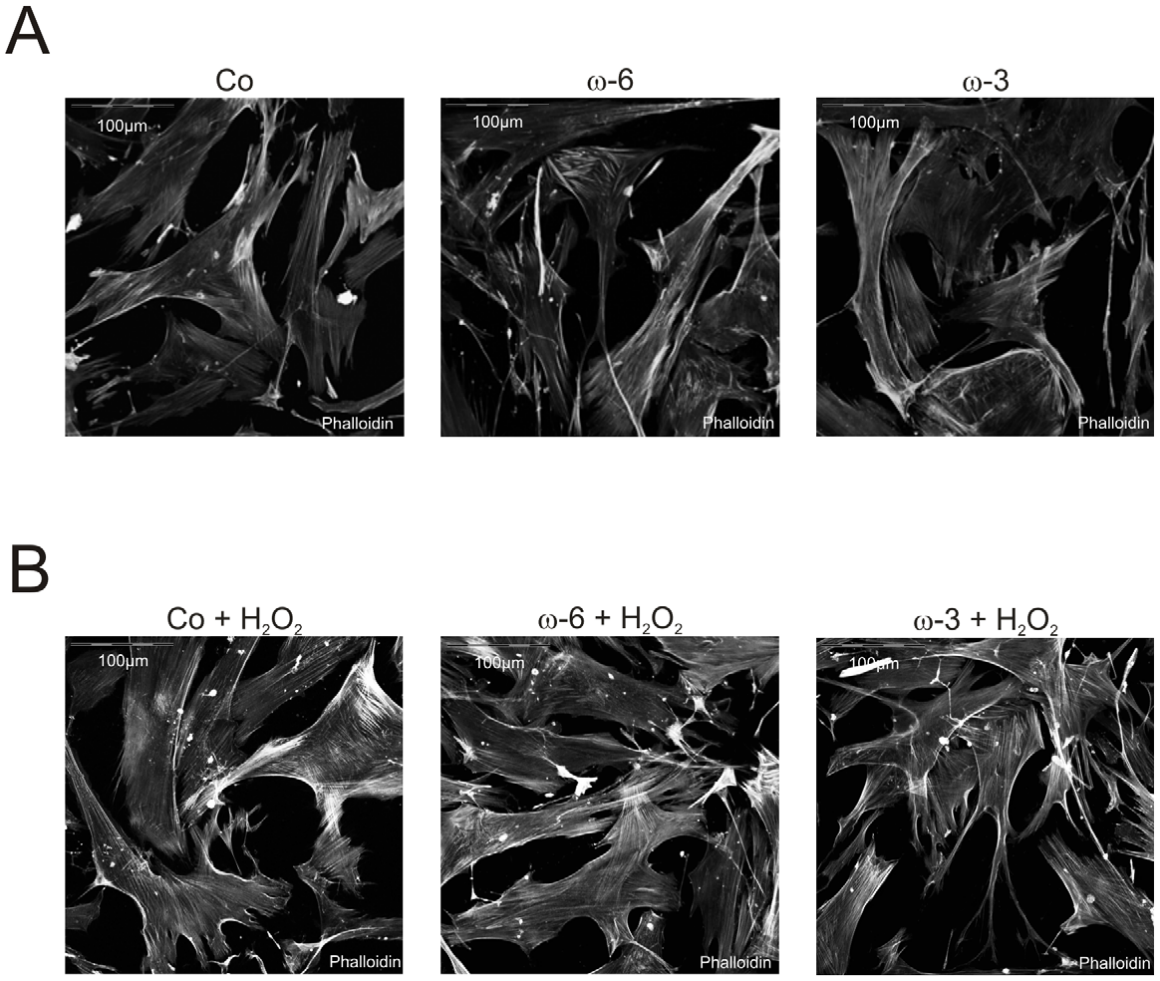
F-actin labelling. Phalloidin labeling of the F-actin cytoskeleton in controls, ω-6 and ω-3 supplemented hTM (**A**) before and (**B**) after H_2_O_2_ exposition; scale bar: 100 µm.

Phalloidin labeling of F-actin revealed no explicit differences between controls and fatty acid supplemented cells with respect to formation of cross-linked actin networks (CLANs; Fig. 7A). It appeared that ω-6 treated cells tended to form increased numbers of stress fibers than the controls as well as ω-3 treated cells (Fig. 7A). H_2_O_2_ exposure promoted accumulation of stress fibers and CLAN formation (Fig. 7B) in the controls. In ω-6 preincubated cells, CLAN formation and stress fibre accumulation appeared even more pronounced (Fig. 7B) than in the stimulated controls and in ω-3 pre-treated cells. ω-3 pre-treated cells showed a similar frequency of CLANs and stress fibres as the unstimulated controls (Fig. 7B).

## Discussion

The current picture of glaucoma pathogenesis suggests that combinatory events act synergistically on the basis of an individual predisposition leading to the onset of the disease [34]. One factor in the focus of investigative glaucoma research is oxidative stress [35], as one of the main problems in glaucoma is an imbalance of ROS formation and decomposition [1] resulting in ROS accumulation [15], [19], [20] as well as general reduction of anti-oxidant capacities. TM cells were shown to be especially sensitive to oxidative stress [8], [19] and typical responses affect ECM synthesis [6], and basic cellular processes, like cell cycle control, proliferation, apoptosis and cellular metabolism [36], [37]. In this respect, prophylactic intake of dietary supplements with alleged antioxidant capacities is recommended for protection from deleterious effects of oxidative stress and prevention of glaucoma thereby.

The aim of this study was to test ω-3 and ω-6 fatty acids for their abilities to antagonize H_2_O_2_ induced glaucomatous effects on cellular activity, proliferation, stress response and ECM synthesis using an in-vitro model based on primary human TM cells.

We found that ω-6 inhibited the normal increase of metabolic activity and proliferation during cultivation that was observed in controls and ω-3 supplemented cells. This suggests an anti-proliferative, cytostatic capacity of ω-6, which would agree to reports about rather deleterious effects of excessive amounts of ω-6, including promotion of cardiovascular disease, inflammation, autoimmune diseases or cancer [26], [27], [28], [38], [39]. Though, extended studies evaluating cell cycle and cell death are necessary to make concrete assertions. In respect of their capacities to prevent TM cells from H_2_O_2_ mediated reductions of metabolism and proliferation, we also found differences for ω-6 and ω-3 fatty acids. BrdU incorporation was efficiently stabilized by both, but only ω-3 rendered hTM unsusceptible against H_2_O_2_ mediated reduction of mitochondrial activity.

Taken together, our data indicate inhibitory side effects of ω-6 on metabolism and proliferation and a limited effectiveness in prevention from oxidative stress. In conclusion, ω-3 appeared to be more beneficial for cellular protection.

Another cellular response to elevated oxidative stress levels is an augmented synthesis of Hsps, which constitute active components of cellular protection and rescue mechanisms [6], [40], [41], [42]. Here we analyzed two representatives of this protein family, Hsp27 and Hsp90, which were found to be strongly expressed in glaucomatous tissues, particularly in the TM. Hsp27 is considered an early marker of cellular stress responses, and is alleged to be especially protective against oxidative damage. Moreover, it was shown that Hsp27 conveys an anti-apoptotic effect by modulation of the nuclear factor (NF) κB-pathway. Hsp90 is also activated by oxidative stress and acts as an essential chaperone maintaining protein stability [42], including transcription factors that regulate anti-apoptotic signaling-pathways.

Our data suggest that Hsp27 is not a direct target of ω-6 and ω-3 fatty acids, as the observed regulations of the mRNA, although statistically significant, did not manifest on the protein level. Hsp90 in contrast, was strongly activated on the RNA level, and also significantly increased on protein level by both supplements. Based on the known functions of Hsp90, this could be interpreted as a kind of cellular alert condition, with a constantly activated mild stress response rendering the cells prepared for potential exogenous threats. In the context of glaucoma prevention, this would be favorable and argue for beneficial effects of ω-6 and ω-3 fatty acids in respect of stress control. Partial support is also added by our data on NFκB and Interleukin synthesis. One feature of oxidative stress is the onset of inflammatory processes that promote disease progression in many neurodegenerative conditions. Key players in this processes are NFκB, regulating expression/secretion of pro-inflammatory cytokines [43]. Here we found that fatty acids appeared to abolish the H_2_O_2_ mediated stimulation of nuclear NFκB, which therefore could be interpreted as an anti-inflammatory effect. Also the stimulation of IL-6, a key pro-inflammatory interleukin and circumscribed oxidative stress marker [44], was repressed by ω-3 and ω-6, for the former even to a significant extent. However, our data on IL-1α and IL-8 did not indicate any effect of fatty acids. Moreover, the observed changes in protein levels were rather subtle, so we cautiously propose a beneficial effect, denoting that this hypothesis requests extended experimental approaches in future studies.

Another typical hallmark of the glaucomatous TM is the accumulation of extracellular material in consequence of an increased synthesis and concurrent repression of proteolytic degradation. It has been already shown that oxidative stress induces synthesis of various ECM components [5], [35] and accordingly we detected an increase of FN, PAI-1 and CTGF mRNA and protein upon H_2_O_2_ exposition. The most obvious effects were observed for FN, which was strongly increased in the medium of ω-6 supplemented and even more increased when the cells were additionally exposed to H_2_O_2_, indicating a potential synergistic effect. CTGF synthesis was also stimulated by ω-6, although significantly less than FN. Notably, there were no indications for a synergistic effect of ω-6 and H_2_O_2_. ω-3 fatty acid did not have significant effects on FN and CTGF expression, but antagonized the H_2_O_2_ mediated stimulation of both proteins. An obvious effect, however, was detected on PAI-1 expression, which was activated by ω-3. Notably, exposition to H_2_O_2_ resulted in lower PAI-1 expression than in the corresponding controls, indicating a similar antagonizing effect as observed with CTGF and FN. Summarized, it appeared that ω-6 fatty acid alone seemed to stimulate ECM synthesis and ω-3 fatty acid seemed to prevent ECM degradation via activation of PAI-1, both effects that would favor ECM accumulation in the context of glaucoma disease. Paradoxically, both fatty acids seem to have the ability to antagonize the H_2_O_2_ mediated stimulations, thus indicating an overall protective effect.

Morphologically, we observed an increased formation of CLANs and intracellular stress fibers after H_2_O_2_ stimulation, a frequent finding in glaucomatous TM cells [5], [45], [46], [47], [48], [49], [50]. Here, only preemptive application of ω-3 had a preventive effect on formation of these stress indicators.

To sum up, ω-6 was efficient in preventing H_2_O_2_ mediated anti-proliferative effects, but displayed a repressive effect on mitochondrial activity and proliferation. For ω-3, we observed no negative side effects but an effective potential to prevent H_2_O_2_ mediated anti-proliferative/-metabolic effects. Both agents induced Hsp90, which can be interpreted in terms of a cellular precaution to forthcoming insults. Considering matrix synthesis, both fatty acids were pro-fibrotic, but still could antagonize H_2_O_2_ stimulation. Lastly, ω-3 was effective in prevention from CLAN and stress fiber formation.

Based on this, we conclude that ω-3 to seems be the more beneficial fatty acid, whereas ω-6 appears more critical and not unreservedly recommendable. This agrees with reported cytotoxic side effects of high-dose ω-6 [26], [27], [28], [38], [39], [51]. Future studies including other vulnerable ocular cell types will have to prove if preemptive dietary with ω-3 helps to prevent deleterious effects of oxidative stress in glaucoma and other age-associated degenerative diseases, and will have to further challenge the eligibility of ω-6 as a protective nutritional supplement.
